# Sleep and Cognition in Community-Dwelling Older Adults: A Review of Literature

**DOI:** 10.3390/healthcare3041243

**Published:** 2015-12-04

**Authors:** Glenna S. Brewster, Miranda Varrasse, Meredeth Rowe

**Affiliations:** 1 College of Nursing, University of South Florida, 12901 Bruce B. Downs Blvd., MDC Box 22, Tampa, FL 33612, USA; E-Mail: mrowe1@health.usf.edu; 2 School of Medicine, University of Pennsylvania, 3624 Market Street, Suite 205, Philadelphia, PA 19104, USA; 3 School of Nursing, University of Pennsylvania, Claire M. Fagin Hall, 418 Curie Boulevard, Philadelphia, PA 19104, USA; E-Mail: mvarr@nursing.upenn.edu

**Keywords:** older adults, sleep parameters, cognition, attention, executive function, verbal fluency, memory

## Abstract

Changes in sleep and cognition occur with advancing age. While both may occur independently of each other, it is possible that alterations in sleep parameters may increase the risk of age-related cognitive changes. This review aimed to understand the relationship between sleep parameters (sleep latency, wake after sleep onset, sleep efficiency, sleep duration, general sleep complaints) and cognition in community-dwelling adults aged 60 years and older without sleep disorders. Systematic, computer-aided searches were conducted using multiple sleep and cognition-related search terms in PubMed, PsycINFO, and CINAHL. Twenty-nine manuscripts met the inclusion criteria. Results suggest an inconsistent relationship between sleep parameters and cognition in older adults and modifiers such as depressive symptoms, undiagnosed sleep apnea and other medical conditions may influence their association. Measures of sleep and cognition were heterogeneous. Future studies should aim to further clarify the association between sleep parameters and cognitive domains by simultaneously using both objective and subjective measures of sleep parameters. Identifying which sleep parameters to target may lead to the development of novel targets for interventions and reduce the risk of cognitive changes with aging.

## 1. Introduction

Alterations in sleep and cognition are associated with advancing age [1,2,3]. Approximately 50% of older adults report that they experience sleep problems [4]. Specifically, older adults report subjective reductions in total sleep time (TST) and sleep efficiency (SE) as well as increases in sleep latency (SL) and wake after sleep onset (WASO) [4]. Changes in sleep parameters add complexity to age-related cognitive changes, as sleep is necessary for healthy brain and bodily function and repair [5,6]. Sleep disturbances may contribute to inadequate central nervous system restoration [7] with the potential to impair cognitive function. There is extensive literature on the role of sleep in memory consolidation [8] but two recent reviews have concluded that the association between sleep and cognition is inconsistent across studies [9,10]. This review is broader in scope in that it specifically examines the associations between specific sleep parameters and the domains of and global cognition in community-dwelling older adults with no complaints of insomnia or other sleep disorders. It is important to determine which sleep parameters have the greatest effect on cognition so that researchers can identify modifiable risk factors for cognitive impairment and know which parameters to target for novel interventions that can potentially decrease the risk for cognitive impairment [10,11].

The objective for this exploratory review of sleep parameters and cognition in community-dwelling adults 60 years and older was to understand the relationships between sleep parameters (sleep latency, wake after sleep onset, sleep efficiency, sleep duration, and general sleep complaints), and the domains of cognition (Executive Function, Attention, Episodic Memory, Working Memory, Processing Speed) and global cognition.

## 2. Methods

Systematic, computer-aided searches were conducted using PubMed, PsycINFO, and CINAHL with the following terms: “cognitive”, “cognition”, “older”, “sleep”, “attention”, “episodic memory”, “executive function”, “processing speed”, “verbal fluency”, and “working memory”. No limits were applied. Reference lists of original and review articles were examined to identify additional relevant publications. To qualify for inclusion in the review, the studies had to include participants who had a mean age of 60 years or older, with no complaints of insomnia, sleep disorders, or cognitive impairment at baseline, and were living independently in the community. The articles also had to report outcome measures of cognition and/or cognitive impairment, have predictor variables of subjective or objective sleep parameters, report original quantitative analyses, and be published in a peer-reviewed journal.

**Figure 1:**
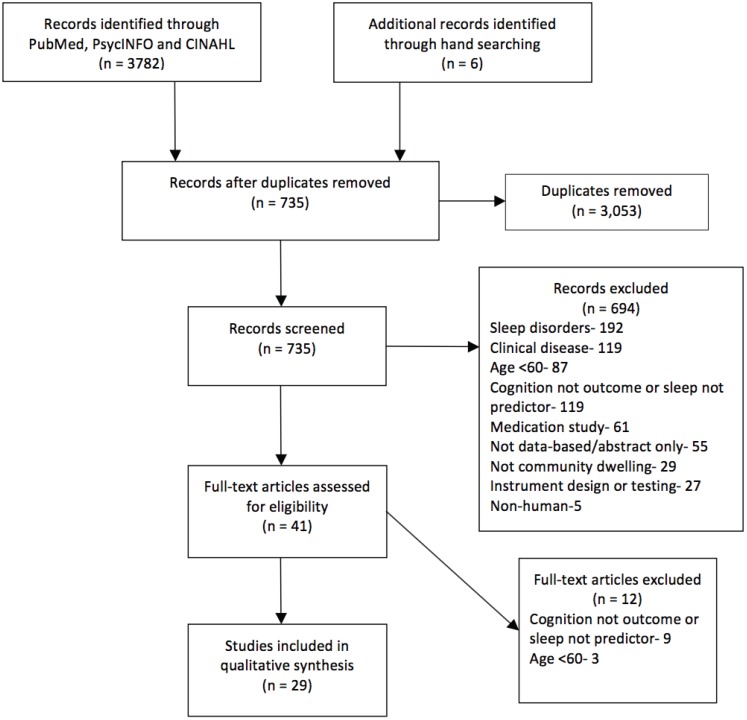
Flow diagram of literature review search for sleep and cognition in older adults.

After removing 3053 duplicates, 735 articles were screened for relevance (See Figure 1). Titles were reviewed and excluded if the title or abstract included a population other than older adults; a sample with sleep disorders; a control for clinical groups; contained the words hospitalized or inpatient; were disease or medication specific; did not contain human subjects; were primarily focused on tool development or measurement testing; were specific to sleep architecture; or did not contain sleep parameters as the dependent variable and cognition as independent variable. Forty-one full text articles were assessed and 12 were excluded based on age <60 or one of the aforementioned sleep parameters was not a predictor of cognition. Twenty-nine articles were identified as fulfilling the inclusion criteria and were included in the review. It must be noted that Blackwell, *et al.* [12] and Blackwell, *et al.* [13] used data from the Study of Osteoporotic Fractures and Devore, *et al.* [14] and Tworoger, *et al.* [15] used data from the Nurses’ Health Study. Articles were reviewed by two authors and discrepancies were discussed. A review of literature table was completed to report the findings of the studies (Table 1).

**Table 1:** Table displaying the design, sample characteristics, results, and exclusion criteria of the studies in the review.

Author, Year Country	Design	Sample Characteristics	Results	Exclusion Criteria/Statistical Adjustment
Ramos *et al.*, (2013) [11] USA	Cross-Sectional	*N*—927 Mean Age—75 % Female—61	TST: Long sleep (≥9 h) inversely associated with MMSE score and short sleep (˂6 h) not associated with MMSE score.	Statistical: Demographics, vascular factors, medications, risk for SDB, depression, alcohol consumption
Blackwell *et al.*, (2006) [12] USA	Cross-Sectional	*N*—2932 Age—83.5 (±3.7) % Female—100	SL: Longer SL was significantly associated with worse global cognition, attention, and executive function. WASO: Longer WASO was significantly associated with worse global cognition, attention, and executive function. SE: Lower SE was significantly associated with worse global cognition, attention, and executive function. TST: TST was significant associated with worse global cognition but was not associated with executive function or attention.	Statistical: Age, race, depression, education, BMI, health status, Hx. of stroke, Hx. of hypertension, IADL impairments, smoking, alcohol use, caffeine intake, antidepressant use, physical activity
Blackwell *et al.*, (2011) [13] USA	Cross-Sectional	*N*—3132 Mean Age—76.4 % Female—0	WASO: Longer objective WASO was associated with poorer global cognition, attention, and executive function. SE: Lower objective SE modeled continuously was associated with poorer attention and executive functioning but not global cognition. TST: Objective long sleep duration was associated with global cognitive functioning but not attention and executive function. Objective short sleep was not associated with global cognition, attention, or EF. Subjective short sleep (<5 h) and long sleep (>8 h) duration were associated with lower levels of global cognition. Long sleep, not short, was associated with poorer attention and executive function. The association between long sleep and global cognition, attention, and executive function disappeared after adjustment with WASO. General Sleep Problems: PSQI (>5) was not associated with global cognitive function, attention, or executive function.	Statistical: Age, race, clinic, education, depression, BMI, number of IADLs, comorbidities, antidepressant use, benzodiazepine use, alcohol use, smoking, physical activity, self-reported health status
Devore *et al.*, (2014) [14] USA	Prospective	*N*—15,263 Mean Age—74.2 % Female—100	TST: Short ≤5 h and long ≥9 h were associated with worse verbal fluency, working and episodic memory and global cognition score than those with 7 h sleep. An increase or decrease in sleep duration was associated with worse verbal fluency, working and episodic memory and global cognition scores. No association was found between sleep duration and cognitive decline.	Statistical: Age, education, shift-work history, smoking status, alcohol intake, physical activity, body mass index, history of high blood pressure, medical outcomes study mental health score, living alone, tranquilizer use
Tworoger *et al.*, (2006) [15] USA	Cross-Sectional Longitudinal (2 years)	*N*—1844 Mean age—74.1 % Female—100	TST: Cross sectionally, short sleep (≤5 h) but not long sleep (≥9 h) duration was associated with an increased risk of global cognitive impairment, and verbal fluency but not episodic memory. Longitudinally (2 years), neither short nor long sleep duration was associated with global cognition, episodic memory, or verbal fluency. General Sleep Problems: Cross-sectionally but not longitudinally, persons who had regular difficulties falling or staying were at an increased risk for poorer global cognitive impairment compared to those with occasional or rare sleep difficulties. There were no cross-sectional or longitudinal associations between sleep difficulties and episodic memory or verbal fluency.	Exclusion Criteria: Taking antidepressants, physician-diagnosis of depression, diagnosis of stroke Statistics: Age, education, smoking status, physical activity, HTN, living status, alcohol consumption, mental health index, use of tranquilizers
Lambaise *et al.*, (2014) [16] USA	Cross-sectional	*N*—121 Mean Age—73.3 % Female—100	SE: Lower objective SE was associated with poorer attention, executive function, and processing speed but not verbal fluency. Subjective SE not associated with attention, executive function, processing speed and verbal fluency. TST: Subjective shorter sleep time was associated with better executive function. Subjective and objective TST were not associated with attention, executive function, processing speed and verbal fluency.	Statistical: Education, race, BMI, depressive symptoms, height, weight, medication use, current hypertension, sleep medication use
Schmutte *et al.*, (2007) [17] USA	Cross-Sectional	*N*—375 Mean Age—79.6 % Female—64.3	SL: Persons with longer SL performed significantly worse on measures of attention, working memory, verbal fluency, and processing speed. SL was not associated with episodic memory. After statistical adjustment, longer SL was associated with only verbal fluency. TST: In univariate analyses, short sleep (˂6 h) and long sleep (˃9 h) duration were not associated with episodic memory, attention, working memory, verbal fluency, or processing speed. ANCOVA analyses for episodic memory indicate an association with longer sleep duration (˃9 h).	Statistical: Depression, age, education, medical comorbidities, physical morbidity, hypnotic use
St. Martin *et al.*, (2012) [18] France	Cross-Sectional	*N*—272 Mean Age—74.8 % Female—71	SL: SL was not associated with any of the 7 cognitive function measures. TST: TST was not associated with any of the 7 cognitive function measures. General Sleep Problems: Higher PSQI total scores were correlated with a poorer global cognitive function, shorter working memory, and worse attention span. Poorer sleep quality associated with shorter working memory and poorer delayed episodic memory.	Exclusion Criteria: MI, heart failure, stroke, previous dementia, neurological D/O, initiation of CPAP for OSA, diagnosis of a new neurological D/O Statistical: Gender, AHI, anxiety, depression, use of sleep meds
Nebes *et al.*, (2009) [19] USA	Cross-sectional	*N*—157 Mean Age—72.2 % Female—Not provided	SL: Longer SL was associated with poorer global cognitive function but not associated with measures of attention, working memory, processing speed, executive function, and episodic memory. SE: Lower SE was associated with poorer global cognitive function and working memory (N-Back) but not associated with other measures of working memory, processing speed, executive function, and episodic memory. TST: Sleep duration was not associated with any of the cognitive function measures. General Sleep Problems: Higher PSQI scores were associated with poorer global cognitive function, a test of executive function(TMT-B), attention (TMT-B), and working memory (N-Back) but not other tests of executive function, processing speed, episodic memory, and working memory.	Exclusion Criteria: No CNS pathology, substance abuse, taking prescription psychoactive medication, no diagnosis of major depression in last five years or GDS score >15 Statistical: Total depressive score, risk of cerebrovascular disease, use of sleeping pills and anticholinergic meds
Miyata *et al.*, (2013) [20] Japan	Cross-Sectional	*N*—78 Mean Age—72.2 % Female—79.5	SL: SL not associated with working memory or attention. WASO: WASO not associated with working memory or attention. SE: Lower SE was significantly associated with worse working memory but not attention. TST: Accuracy of 0-back was different for those with ˂5 h than those with ˃7 h. No difference was seen between the participants with short and long sleep duration with accuracy on the 1-back test and the attention measure. General Sleep Problems: Global sleep quality was not associated with working memory and attention.	None provided
Chang-Quan, Bi-Rong & Yan, (2012) [21] China	Cross-Sectional	*N*—660 Mean Age—93.5 % Female—67.3	SL: Longer SL was correlated with cognitive impairment. SE: Lower SE was correlated with cognitive impairment. General Sleep Problems: Poor sleep quality increased the risk for cognitive impairment.	Statistical: Age, gender, education level, serum lipid/lipoprotein, BMI, blood pressure, blood glucose level, smoking habit, alcohol consumption, tea consumption, exercise
Auyeung *et al.*, (2011) [22] China	Cross-Sectional	*N*—2945 Age—73.9 (±5.0) % Female—40.8	SL: A higher MMSE score was significantly associated with fewer reports of prolonged SL before and after statistical adjustment. TST: Longer nocturnal TST (>7 h) was significantly associated with lower general cognition. No association between global CF and short sleep duration (4 h to 7.9 h). General Sleep Problems: A higher MMSE score was significantly associated with less chronic sleep complaints in the univariate but not multivariate analyses.	Exclusion Criteria: Cognitively incompetent to give informed consent, medical conditions that made them unlikely to complete the study Statistical: Age, gender, MMSE score, education, smoking, alcohol, tea and coffee consumption, habitual smoking, depression (GDS ≥8), use of psychotropic meds, dx of HTN, diabetes, stroke, CHD, COPD
Keage *et al.*, (2012) [23] UK	Cross-Sectional Longitudinal (2 and 10 years)	*N*-Baseline—2041 2 years—1658 10 years—663 Median age—75 % Female—53	SL: SL was not cross-sectionally associated with cognitive impairment or predicted cognitive decline after 2 or 10 years. WASO: WASO was not cross-sectionally or longitudinally associated with cognitive impairment. TST: Both short (≤6.5 h) and long (≥9 h) sleep duration were not cross-sectionally associated with global cognitive impairment. Short sleep duration was associated with incident cognitive impairment over 10 years. Long sleep duration did not predict risk for cognitive impairment at years two and 10. General Sleep Problems: General sleep problems were not cross-sectionally or longitudinally associated with cognitive impairment.	Statistical: MMSE ≤21 at baseline, sex, age at baseline, BMI classification, education
Potvin *et al.*, (2012) [24] Canada	Longitudinal (1 year)	*N*—1664 Mean Age Male—72.7 Female—73.9 % Female—69.7	SL: SL was not associated with incident cognitive decline. SE: In women, SE was not associated with incident cognitive decline. In men, sleep efficiency predicted incident cognitive decline after one year. TST: Short sleep duration (≤5 h) was associated with incident cognitive decline in men and not women. In women and not men, long sleep duration (≥9 h) was associated with incident cognitive impairment over one year. General Sleep Problems: In women but not men, PSQI sleep disturbance score was associated with general cognitive decline one year later. In men but not women, global sleep quality score was associated with incident cognitive decline after one year.	Exclusion Criteria: Dementia, Cerebrovascular disease, Brain trauma/tumor/infections, Parkinson’s disease, Epilepsy, Schizophrenia and other forms of psychosis, Baseline MMSE score below the 15th percentile Statistical: Age, education, baseline MMSE score, anxiety, depressive episode psychotropic drug use, cardiovascular conditions score, chronic diseases
Jaussent *et al.*, (2012) [25] France	Longitudinal (8 years)	*N*—4894 Mean Age—Not provided % Female—57	SOL: SOL not associated with cognitive decline. WASO: As WASO increased, the risk of experiencing cognitive decline increased. General Sleep Problems: Sleep quality not associated with cognitive decline.	Statistical: Study center, sex, age, educational level, MMSE score at baseline, prescribed sleep meds, insomnia severity
Wilckens *et al.*, (2014) [26] USA	Cross-Sectional	*N*—53 Mean Age—62.68 % Female—Not provided	WASO: Lower WASO was associated with better executive function, verbal fluency, and episodic memory, but not working memory or processing speed. TST: Neither total, long, nor shoet sleep duration were associated with executive function, episodic memory, working memory, verbal fluency or processing speed.	Exclusion Criteria: Self-reported diagnosis of depression, current psychiatric medication use, dependence on drugs or alcohol, diagnosis of a neurodegenerative disease Statistical: Sex, education
McCrae *et al.*, (2012) [27] USA	Cross-Sectional	*N*—72 Mean Age—70.2 % Female—Not provided	TST: TST did not predict executive functioning or processing speed. General Sleep Problems: Total wake time did not predict executive functioning but significantly predicted processing speed.	Exclusion Criteria: Medical and neurological disorder, psychopathology, sleep disorders (OSA, RLS), MMSE lower than 23, severe depressive symptoms, suspected SDB, missing more than seven days of sleep data
Benito-Leon, Louis & Bermejo-Pareja, (2013) [28] Spain	Longitudinal (3 years)	*N*—2715 Age—72.9 (±6.1) % Female—56.9	TST: At baseline, short sleep (≤5 h) global CF score was significantly different than reference (6–8 h) group but long sleep (≥9 h) global CF score not significantly different. Longitudinally, change in global CF associated with long sleep but not short sleep. Rate of cognitive decline not significantly different between short sleep and reference but significantly different between long sleep and reference groups. Long sleepers were 1.3 times more likely to have cognitive decline than reference group. Short sleepers’ odds of having cognitive decline similar to reference group.	Exclusion Criteria: Age, gender, geographical area, educational level, diabetes mellitus, chronic obstructive pulmonary disease, depressive symptoms, antidepressant use, medications with central nervous system effects
Virta *et al.*, (2013) [29] Finland	Longitudinal (22.5 years)	*N*—2336 Mean Age—74.4 % Female—47.9	TST: Short sleep duration (<7 h) and long sleep duration (>8 h) associated with poorer cognition. General Sleep Complaints: Poor sleep quality associated with poorer cognition.	Statistical: Snoring, use of hypnotics and tranquilizers, age, educational level, life satisfaction, obesity, hypertension, leisure time physical activity, alcohol consumption, binge drinking, APOE genotype
Loerbroks *et al.*, (2010) [30] Germany	Cross-Sectional Longitudinal (8.5 years)	*N*—695 Mean Age—72.1 % Female—59	TST: Short (≤6 h) and long (≥9 h) sleep duration were not cross-sectionally or longitudinally associated with global cognitive function. After statistical adjustment, a decline in sleep duration did not predict global cognitive impairment but an increase in sleep duration was associated with a two-fold increase in global cognitive impairment after 8.5 years.	Exclusion Criteria: Depression, taking mood enhancing drugs Statistical: Age, gender, educational level, physical activity, alcohol consumption, body mass index, smoking status, use of sleep medication, depressive symptoms at the time of testing
Xu *et al.*, (2010) [31] China	Cross Sectional	*N*—28,670 Mean Age—62 % Female—72.5	TST: Short TST(3–4 h and 5 h) and long TST (more than 10 h) were associated with worse episodic memory and global cognition.	Exclusion Criteria: self-reported mental illness or neurological disease, extremely short or long sleep duration Statistical: Age, sex, employment, occupation, education, smoking, drinking, physical activity, tea consumption, self-rated health, waist circumference, cholesterol, fasting plasma glucose, systolic blood pressure, sleeping duration, napping, insomnia, feeling tired in the morning
Ohayon & Vecchierini, (2002) [32] France	Cross-Sectional	*N*—1026 Mean age—Not provided % Female—59.8	TST: Short sleep time (<7 h), but not long sleep duration (>8.5 h), was associated with attention-concentration deficits and difficulties in orientation for persons but not praxis, delayed recall, difficulties in temporal orientation, and prospective memory using the McNair Scale. Neither long nor short sleep duration was associated with MMSE.	Statistical: Age, sex, physical activity, occupation, organic diseases, use of sleep or anxiety medications, psychological well being
Faubel *et al.*, (2009) [33] Spain	Cross-Sectional	*N*—3212 Age—71.6 % Female—52.6	TST: Long sleep duration (>10 h) was associated with an increased risk for cognitive impairment. Short sleep duration (<7 h) was not associated with an increased risk of cognitive impairment. As TST increased from 7 h to 11 h, cognition progressively worsened.	Exclusion Criteria: Diagnosis of depression, extreme sleep duration <4 h or >17 h, dementia diagnosis Statistical: Age sex, physical activity, tobacco use, alcohol consumption, coffee consumption, educational level, SF-36 mental and physical summary scores, night time awakening, BMI, chronic diseases, anxiolytic and medical drug use, HTN, antihypertensive meds, number of social ties, head of family’s work status
Sampaio *et al.*, (2012) [34] Japan	Cross-Sectional	*N*—145 Mean Age—73 % Female—53.1%	General Sleep Problems: Significant difference reported between good and poor sleepers on global cognition.	Exclusion Criteria: MMSE ≤21, uncontrolled cardiovascular, pulmonary, or metabolic diseases, surgery or forced bedrest in the past three months, current treatment for cancer, orthopedic condition that could restrict ADLs Statistical: Sex, education, living situation, work, financial satisfaction, smoking, alcohol, number of consultations in six months, number of medications, morbidities, comorbidities and regular physical activity categories.
Lim *et al.*, (2013) [35] USA	Prospective Longitudinal (6 years)	*N*—737 Age—81.6 % Female—76	General Sleep Problems: Increased sleep fragmentation associated with lower baseline global cognition and a more rapid rate of global cognitive decline. Persons with high sleep fragmentation had an increased risk of developing Alzheimer’s disease.	Statistical: Age, sex, education, time
Foley *et al.* (2001) [36] USA	Longitudinal (3 year)	*N*—2346 Mean Age—76.6 % Female—0	General Sleep Problems: Having trouble falling asleep or waking up too early and being unable to fall asleep again at baseline was not predictive of global cognition 3 years later.	Exclusion Criteria: Diagnosis of dementia Statistical: Age, education, APOE, CASI score, depressive symptoms, hours of sleep, daytime napping, coronary heart disease, history of stroke
Gamaldo, Allaire & Whitfield, (2008) [37] USA	Cross-Sectional	*N*—174 Mean Age—72.7 % Female—70.7	General Sleep Problems: There was a negative association between trouble falling asleep and working memory. There were no significant associations between trouble falling sleep and global cognition or episodic memory. Trouble falling asleep predicted working memory but not global cognition or episodic memory after statistical adjustment.	Statistical: Age, gender, education, depression, health, income
Zimmerman *et al.*, (2012) [38] USA	Cross-Sectional	*N*—549 Mean Age—79.7 % Female—62.1	General Sleep Problems: General sleep onset/maintenance difficulties were not associated with any of the cognition measures.	Exclusion Criteria: Visual and auditory impairment, active psychiatric symptoms, dementia, amnestic MCI Statistical: Age, gender, ethnicity, depression, cardiovascular history
Sutter *et al.*, (2012) [39] Zurich	Cross-Sectional	*N*—96 Mean Age—72 % Female—57	General Sleep Problems: Poor sleep quality was negatively associated with executive function, verbal fluency, and attention at higher levels of depression. Sleep quality was not associated with processing speed and episodic memory.	Exclusion Criteria: Parkinson’s disease, clinical significant depressive symptoms, use of antidepressants, Statistical: Age, sleep medications

KEY: AHI—Apnea Hypopnea Index; ANCOVA—Analysis of Covariance; APOE—Apolipoprotein E; Att.—Attention; BMI-Body Mass Index; CASI—Cognitive Abilities Screening Instrument; CHD—Coronary Heart Disease; CF—Cognitive Function; CNS—Central Nervous System; COPD—Chronic Obstructive Pulmonary Disease; CPAP—Continuous Positive Airway Pressure; D/O—Disorder; EF—Executive Function; EM—Episodic Memory; FU—Follow-up; GDS—Geriatric Depression Scale; H—Hours; Hx.—History; HTN—Hypertension; IADL—Instrumental Activities of Daily Living; MCI—Mild Cognitive Impairment; MMSE—Mini-Mental State Examination; OSA—Obstructive Sleep Apnea; PS—Processing Speed; PSQI—Pittsburgh Sleep Quality Index; RLS—Restless Legs Syndrome; SDB—Sleep Disordered Breathing; SE—Sleep Efficiency; SF-36—Short-Form-36; SL—Sleep Latency; TMT-B—Trail Making Test B; TST—Total Sleep Time; VF—Verbal Fluency; WASO—Wake After Sleep Onset; WM—Working Memory.

## 3. Results

Investigator-developed questionnaires, or proxy measures of sleep, were used to subjectively measure sleep in the majority of the studies. Also, each of these measures evaluated the sleep parameters using different questions. The Pittsburgh Sleep Quality Index (PSQI) [40] was another frequently used instrument for subjective sleep assessment. Sleep diaries were used in three of the studies. Of the studies that used actigraphy to measure sleep objectively, Blackwell, *et al.* [13] and Lambiase, *et al.* [16] used a sleep diary concurrently (See Table 2).

**Table 2:** Table displaying the measures used to assess sleep.

Subjective	Objective
**Investigator-Developed Sleep Questionnaire**	**Actigraphy**
Ramos *et al.*, (2013) [11]; Devore *et al.*, (2014) [14]; Tworoger *et al.*, (2006) [15]; Schmutte *et al.*, (2007) [17]; Auyeung *et al.*, (2013) [22]; Keage *et al.*, (2012) [23]; Jaussent *et al.*, (2012) [25]; Virta *et al.*, (2013) [29]; Loerbroks *et al.*, (2010) [30]; Xu *et al.*, (2010) [31]; Faubel *et al.*, (2009) [33]; Sampaio *et al.*, (2012) [34]; Lim *et al.*, (2013) [35]; Foley *et al.*, (2001) [36]; Gamaldo, Allaire & Whitfield, (2008) [37]	Blackwell *et al.*, (2006) [12]; Blackwell *et al.*, (2011) [13]; Lambaise *et al.*, (2014) [16]; Miyata *et al.*, (2013) [20]; Lim *et al.*, (2013) [35]
**Pittsburgh Sleep Quality Index**	**SensaWear**
Blackwell *et al.*, (2011) [13]; St. Martin *et al.*, (2012) [18]; Nebes *et al.*, (2009) [19]; Miyata *et al.*, (2013) [20]; Chang-Quan, Bi-Rong & Yan, (2012) [21]; Potvin *et al.*, (2012) [24]; Sutter *et al.* (2012) [39]	Wilckens *et al.*, (2014) [26]
**Sleep Diary**	
Blackwell *et al.*, (2011) [13]; Lambaise *et al.*, (2014) [16]; McCrae *et al.*, (2012) [27]	
**Sleep-EVAL System**	
Blackwell *et al.*, (2011) [13]; Lambaise *et al.*, (2014) [16]; McCrae *et al.*, (2012) [27]	
**Medical Outcomes Study Sleep Scale**	
Zimmerman *et al.*, (2012) [38]	

There was also a large variety of tests and instruments that were used to assess the domains of cognition and global cognition (See Table 3). The Mini-Mental State Exam (MMSE) [41] was used in more than half of the studies to assess global cognition. In addition, some of the tests in this study overlapped domains, making it difficult to demonstrate clear differences in the association between sleep parameters and specific domains of cognition. For example, Trail Making Part B is used to measure both attention and executive function. However, this is an issue that plagues cognition research [42,43] and is difficult to avoid.

In order to understand the relationship between sleep and cognition, each parameter of sleep and its association with cognition will be discussed in the following section (see Table 4 and Table 5).

**Table 3:** Table displaying the tests used to assess the domains of and global cognition.

Executive Function	Attention	Episodic Memory	Working Memory	Verbal Fluency	Processing Speed	Global Cognition
**German Achievement Measure Test**	**Continuous Performance Test**	**Delayed Recall of the TICS 10 Word List**	**Alpha Span Task**	**Alphabetic/Word Fluency**	**Code Test**	**(Modified) Mini-Mental Status Exam**
Sutter *et al.*, (2012) [39]	Miyata *et al.*, (2013) [20]	Devore *et al.*, (2014) [14]; Tworoger *et al.*, (2006) [15]	Gamaldo, Allaire & Whitfield, (2008) [37]	Lambaise *et al.*, (2014) [16]; St. Martin *et al.*, (2012) [18]; Wilckens *et al.*, (2014) [26]; Zimmerman *et al.*, (2012) [38]; Sutter *et al.*, (2012) [39]	St. Martin *et al.*, (2012) [18]	Ramos *et al.*, (2013) [11]; Blackwell *et al.*, (2011) [13]; Tworoger *et al.*, (2006) [15]; St. Martin *et al.*, (2012) [18]; Chang-Quan, Bi-Rong & Yan, (2012) [21]; Auyeung *et al.*, (2013) [22]; Keage *et al.*, (2012) [23]; Potvin *et al.*, (2012) [24]; Jaussent *et al.*, (2012) [25]; McCrae *et al.*, (2012) [27]; Xu *et al.*, (2010) [31]; Ohayon & Vecchierini, (2002) [32]; Faubel *et al.*, (2009) [33]; Sampaio *et al.*, (2012) [34]; Gamaldo, Allaire & Whitfield, (2008) [37]
**Go/No-Go Task**	**Digit Vigilance Test**	**East Boston Memory Test**	**Benton Visual Retention Test**	**Category Fluency**	**Conceptual and Perceptual Comparison**	**Cognitive Abilities Screening Instrument**
Sutter *et al.*, (2012) [39]	Blackwell *et al.*, (2011) [13]	Devore *et al.*, (2014) [14]; Tworoger *et al.*, (2006) [15]	Jaussent *et al.*, (2012) [25]; St. Martin *et al.*, (2012) [18]	Devore *et al.*, (2014) [14]; Lambaise *et al.*, (2014) [16]; St. Martin *et al.*, (2012) [18]; Schmutte *et al.*, (2007) [17]; Sutter *et al.*, (2012) [39]; Tworoger *et al.*, (2006) [15]; Zimmerman *et al.*, (2012) [38]; Wilckens *et al.*, (2014) [26]	Nebes *et al.*, (2009) [19]	Foley *et al.*, (2001) [36]
**Hayling Test **	**Months Backward**	**Letter Series Task**	**Digit Span Backwards**		**Digit Symbol Substitution**	**Telephone Interview for Cognitive Status**
Nebes *et al.*, (2009) [19]	Schmutte *et al.*, (2007) [17]	McCrae *et al.*, (2012) [27]	Devore *et al.*, (2014) [14]; Tworoger *et al.*, (2006) [15]; Schmutte *et al.*, (2007) [17]; Loerbroks *et al.*, (2010) [30]; Gamaldo, Allaire & Whitfield, (2008) [37]; Zimmerman *et al.*, (2012) [38]		Lambaise *et al.*, (2014) [16]; Schmutte *et al.*, (2007) [17]; Wilckens *et al.*, (2014) [26]; Sutter *et al.*, (2012) [39]	Devore *et al.*, (2014) [14]; Tworoger *et al.*, (2006) [15]; Virta *et al.*, (2013) [29]; Loerbroks *et al.*, (2010) [30]
**Stroop Test**	**Trail Making Test Part A**	**Logical Memory Test**	**Forward Digit Span**		**Symbol Digit Modalities Test**	**Repeatable Battery for the Assessment of Neuropsychological Status**
St. Martin *et al.*, (2012) [18]; Nebes *et al.*, (2009) [19]; Wilckens *et al.*, (2014) [26]	Lambaise *et al.*, (2014) [16]; St. Martin *et al.*, (2012) [18]; Zimmerman *et al.*, (2012) [38]; Sutter *et al.*, (2012) [39]	Nebes *et al.*, (2009) [19]	Gamaldo, Allaire & Whitfield, (2008) [37]		McCrae *et al.*, (2012) [27]	Nebes *et al.*, (2009) [19]
**Trail Making Test Part B**	**Trail Making Test Part B**	**Selective Reminding Test**	**N-Back Test**			**Composite of Tests of Multiple Domains**
Blackwell *et al.*, (2006) [12]; Blackwell *et al.*, (2011) [13]; Lambaise *et al.*, (2014) [16]; St. Martin *et al.*, (2012) [18]; Nebes *et al.*, (2009) [19]; Zimmerman *et al.*, (2012) [38]; Sutter *et al.*, (2012) [39]	Blackwell *et al.*, (2006) [12]; Blackwell *et al.*, (2011) [13]; Lambaise *et al.*, (2014) [16]; St. Martin *et al.*, (2012) [18]; Nebes *et al.*, (2009) [19]; Zimmerman *et al.*, (2012) [38]; Sutter *et al.*, (2012) [39]	Schmutte *et al.*, (2007) [17]; St. Martin *et al.*, (2012) [18]; Zimmerman *et al.*, (2012) [38]	Miyata *et al.*, (2013) [17]; Nebes *et al.*, (2009) [19]; Wilckens *et al.*, (2014) [26]			Lim *et al.*, (2013) [35]
		**Verbal Learning Test**	**Stenberg Working Memory Task**			**Cognitive Difficulties Scale**
		Gamaldo, Allaire & Whitfield, (2008) [37]; Sutter *et al.*, (2012) [39]	Wilckens *et al.*, (2014) [26]			St. Martin *et al.*, (2012) [18]; Ohayon & Vecchierini, (2002) [32]
		**Consortium to Establish a Registry for Alzheimer’s Disease List Memory Test**				**Blessed Information Memory Concentration Test**
		Wilckens *et al.*, (2014) [26]; Xu *et al.*, (2010) [31]				Zimmerman *et al.*, (2012) [38]
						TELE
						Virta *et al.*, (2013) [29]

**Table 4:** Relationship between subjective sleep parameters and the domains of and global cognition.

Sleep Parameters		Executive Function	Attention	Episodic Memory	Working Memory	Verbal Fluency	Processing Speed	Global Cognition
Long Sleep Latency	Sig		Schmutte *et al.*, (2007) [17]*		Schmutte *et al.*, (2007) [17]*	Schmutte *et al.*, (2007) [17]	Schmutte *et al.*, (2007) [17]*	Nebes *et al.*, (2009) [19]; Chang-Quan *et al.*, (2012) [21]; Auyeung *et al.*, (2013) [22]
	NS	St. Martin *et al.*, (2012) [18]; Nebes *et al.*, (2009) [19]	Schmutte *et al.*, (2007) [17]; St. Martin *et al.*, (2012) [18]; Nebes *et al.*, (2009) [19]	Tworoger *et al.*, (2006) [15]; Schmutte *et al.*, (2007) [17]; St. Martin *et al.*, (2012) [18]	Schmutte *et al.*, (2007) [17]; St. Martin *et al.*, (2012) [18]; Nebes *et al.*, (2009) [19]; Miyata *et al.*, (2013) [20]	St. Martin *et al.*, (2012) [18]	Schmutte *et al.*, (2007) [17]; St. Martin *et al.*, (2012) [18]; Nebes *et al.*, (2009) [19]	St. Martin *et al.*, (2012) [18]; Keage *et al.*, (2012) [23]; Potvin *et al.*, (2012) [24]; Jaussent *et al.*, (2012) [25]
Long Wake After Sleep Onset	Sig							Chang-Quan *et al.*, (2012) [21]
	NS							Keage *et al.*, (2012) [23]
Low Sleep Efficiency	Sig							Nebes *et al.*, (2009) [19]; Chang-Quan *et al.*, (2012) [21]; Potvin *et al.*, (2012) [24]*
	NS	Lambaise *et al.*, (2014) [16]; Nebes *et al.*, (2009) [19]	Lambaise *et al.*, (2014) [16]; Nebes *et al.*, (2009) [19]	Nebes *et al.*, (2009) [19]	Nebes *et al.*, (2009) [19]	Lambaise *et al.*, (2014) [16]	Lambaise *et al.*, (2014) [16]; Nebes *et al.*, (2009) [19]	Tworoger *et al.*, (2006) [15]; Potvin *et al.*, (2012) [24]
Short Sleep Duration	Sig		Loerbroks *et al.*, (2010) [30]	Devore *et al.*, (2014) [14]; Xu *et al.*, (2011) [31]	Devore *et al.*, (2014) [14]	Devore *et al.*, (2014) [14]; Tworoger *et al.*, (2006) [15]*		Blackwell *et al.*, (2011) [13]; Devore *et al.*, (2014) [14]; Tworoger *et al.*, (2006) [15]*; Keage *et al.*, (2012) [23]*; Potvin *et al.*, (2012) [24]*; Benito-Leon *et al.*, (2013) [28]*; Virta *et al.*, (2013) [29]; Xu *et al.*, (2011) [31]
	NS	Blackwell *et al.*, (2011) [13]	Blackwell *et al.*, (2011) [13]; Schmutte *et al.*, (2007) [17]	Tworoger *et al.*, (2006) [15]; Schmutte *et al.*, (2007) [17]; Loerbroks *et al.*, (2010) [30]	Schmutte *et al.*, (2007) [17]	Tworoger *et al.*, (2006) [15]; Schmutte *et al.*, (2007) [17]	Schmutte *et al.*, (2007) [17]	Ramos *et al.*, (2013) [11]; Tworoger *et al.*, (2006) [15]; Auyeung *et al.*, (2013) [22]; Keage *et al.*, (2012) [23]; Potvin *et al.*, (2012) [24]; McCrae *et al.*, (2012) [27]; Loerbroks *et al.*, (2010) [30]; Ohayon & Vecchierini, (2002) [32]; Faubel *et al.*, (2009) [33]
Long Sleep Duration	Sig	Blackwell *et al.*, (2011) [13]	Blackwell *et al.*, (2011) [13]	Schmutte *et al.*, (2007) [17]*; Xu *et al.* (2011) [31]				Ramos *et al.*, (2013) [11]; Blackwell *et al.*, (2011) [13]; Auyeung *et al.*, (2013) [22]; Potvin *et al.*, (2012) [24]*; Benito-Leon *et al.* (2013) [28]*; Virta *et al.*, (2013) [29]; Xu *et al.*, (2011) [31]; Faubel *et al.*, (2009) [33]
	NS	Wilckens *et al.*, (2014) [26]; Ohayon & Vecchierini, (2002) [32]	Schmutte *et al.*, (2007) [17]; Loerbroks *et al.*, (2010) [30]	Tworoger *et al.*, (2006) [15]; Schmutte *et al.*, (2007) [17]; Wilckens *et al.*, (2014) [26]; Loerbroks *et al.*, (2010) [30]; Ohayon & Vecchierini, (2002) [32]	Schmutte *et al.*, (2007) [17]; Wilckens *et al.*, (2014) [26]; Loerbroks *et al.*, (2010) [30]	Tworoger *et al.*, (2006) [15]; Schmutte *et al.*, (2007) [17]; Wilckens *et al.*, (2014) [26]; Loerbroks *et al.*, (2010) [30]	Schmutte *et al.*, (2007) [17]; Wilckens *et al.*, (2014) [26]	Tworoger *et al.*, (2006) [15]; Keage *et al.*, (2012) [23]; Potvin *et al.*, (2012) [24]; Benito-Leon *et al.*, (2013) [28]; Loerbroks *et al.*, (2010) [30]
Total Sleep Duration	Sig	Lambaise *et al.*, (2014) [16]	Lambaise *et al.*, (2014) [16]					
	NS	St. Martin *et al.*, (2012) [18]; Nebes *et al.*, (2009) [19]; McCrae *et al.*, (2012) [27]	St. Martin *et al.*, (2012) [18]; Nebes *et al.*, (2009) [19]	St. Martin *et al.*, (2012) [18]; Nebes *et al.*, (2009) [19]	St. Martin *et al.*, (2012) [18]; Nebes *et al.*, (2009) [19]	Lambaise *et al.*, (2014) [16]; St. Martin *et al.*, (2012) [18]	St. Martin *et al.*, (2012) [18]; Nebes *et al.*, (2009) [19]; McCrae *et al.*, (2012) [27]	St. Martin *et al.*, (2012) [18]; Nebes *et al.*, (2009) [19]
General Sleep Problems	Sig	Nebes *et al.*, (2009) [19]*; Sutter *et al.*, (2012) [39]	St. Martin *et al.*, (2012) [18]; Nebes *et al.*, (2009) [19]; Sutter *et al.*, (2012) [39]	St. Martin *et al.*, (2012) [18]*	St. Martin *et al.*, (2012) [18]; Nebes *et al.*, (2009) [19]*; Gamaldo *et al.*, (2008) [37]	Sutter *et al.*, (2012) [39]	McCrae *et al.*, (2012) [27]	Tworoger *et al.*, (2006) [15]*; St. Martin *et al.*, (2012) [18]*; Nebes *et al.*, (2009) [19]; Chang-Quan *et al.*, (2012) [21]; Auyeung *et al.*, (2013) [22]*; Potvin *et al.*, (2012) [24]*; Virta *et al.*, (2013) [29]; Sampaio *et al.*, (2012) [34]
	NS	Blackwell *et al.*, (2011) [13]; St. Martin *et al.*, (2012) [18]; Nebes *et al.*, (2009) [19]; McCrae *et al.*, (2012) [27]; Zimmerman *et al.*, (2012) [38]	Blackwell *et al.*, (2011) [13]; Miyata *et al.*, (2013) [20]; Zimmerman *et al.*, (2012) [38]	Tworoger *et al.*, (2006) [15]; St. Martin *et al.*, (2012) [18]; Nebes *et al.*, (2009) [19]; Gamaldo *et al.*, (2008) [37]; Sutter *et al.*, (2012) [39]	Zimmerman *et al.*, (2012) [38]	Tworoger *et al.*, (2006) [15]; St. Martin *et al.*, (2012) [18]; Zimmerman *et al.*, (2012) [38]	St. Martin *et al.*, (2012) [18]; Nebes *et al.*, (2009) [19]; Sutter *et al.*, (2012) [39]	Blackwell *et al.*, (2011) [13]; Tworoger *et al.*, (2006) [15]; St. Martin *et al.*, (2012) [18]; Auyeung *et al.*, (2013) [22]; Keage *et al.*, (2012) [23]; Potvin *et al.*, (2012) [24]; Jaussent *et al.*, (2012) [25]; Foley *et al.*, (2001) [36]; Gamaldo *et al.*, (2008) [37]; Zimmerman *et al.*, (2012) [38]

KEY: NS—non-significant; Sig—Significant; * Studies with both significant and non-significant results for the same sleep component.

**Table 5:** Relationship between objective sleep parameters and the domains of and global cognition.

Sleep Parameters			Executive Function	Attention	Episodic Memory	Working Memory	Verbal Fluency	Processing Speed	Global Cognition
Long Sleep Latency		Sig	Blackwell *et al.*, (2006) [12]	Blackwell *et al.*, (2006) [12]					Blackwell *et al.*, (2006) [12]
		NS		Miyata *et al.*, (2013) [20]		Miyata *et al.*, (2013) [20]			
Long Wake After Sleep Onset		Sig	Blackwell *et al.*, (2006) [12]; Blackwell *et al.*, (2011) [13]; Wilckens *et al.*, (2014) [26]	Blackwell *et al.*, (2006) [12]; Blackwell *et al.*, (2011) [13]	Wilckens *et al.*, (2014) [26]		Wilckens *et al.*, (2014) [26]		Blackwell *et al.*, (2006) [12]; Blackwell *et al.*, (2011) [13]
		NS		Miyata *et al.*, (2013) [20]		Miyata *et al.*, (2013) [20]; Wilckens *et al.*, (2014) [26]		Wilckens *et al.*, (2014) [26]	
Low Sleep Efficiency		Sig	Blackwell *et al.*, (2006) [12]; Blackwell *et al.*, (2011) [13]; Lambaise *et al.*, (2014) [16]	Blackwell *et al.*, (2006) [12]; Blackwell *et al.*, (2011) [13]; Lambaise *et al.*, (2014) [16]		Miyata *et al.*, ( 2013) [20]		Lambaise *et al.*, (2014) [16]	Blackwell *et al.*, (2006) [12]
		NS		Miyata *et al.*, ( 2013) [20]			Lambaise *et al.*, (2014) [16]		Blackwell *et al.*, (2011) [13]
Sleep Dura-tion	Short	Sig				Miyata *et al.*, (2013) [20]			
		NS	Blackwell *et al.*, (2011) [13]; Wilckens *et al.*, (2014) [26]	Blackwell *et al.*, (2011) [13]; Miyata *et al.*, (2013) [20]	Wilckens *et al.*, (2014) [26]	Miyata *et al.*, (2013) [20]; Wilckens *et al.*, (2014) [26]	Wilckens *et al.*, (2014) [26]	Wilckens *et al.*, (2014) [26]	Blackwell *et al.*, (2011) [13]
	Long	Sig							Blackwell *et al.*, (2011) [13]
		NS	Blackwell *et al.*, (2011) [13]; Wilckens *et al.*, (2014) [26]	Blackwell *et al.*, (2011) [13]; Miyata *et al.*, (2013) [20]	Wilckens *et al.*, (2014) [26]	Miyata *et al.*, (2013) [20]; Wilckens *et al.*, (2014) [26]	Wilckens *et al.*, (2014) [26]	Wilckens *et al.*, (2014) [26]	
	Total	Sig							Blackwell *et al.*, (2006) [12]
		NS	Blackwell *et al.*, (2006) [12]; Lambaise *et al.*, (2014) [16]; Wilckens *et al.*, (2014) [26]	Blackwell *et al.*, (2006) [12]; Lambaise *et al.*, (2014) [16]	Wilckens *et al.*, (2014) [26]	Wilckens *et al.*, (2014) [26]	Lambaise *et al.*, (2014) [16]; Wilckens *et al.*, (2014) [26]	Lambaise *et al.*, (2014) [16]; Wilckens *et al.*, (2014) [26]	
General Sleep Problems		Sig							Lim *et al.*, (2013) [35]
		NS							

KEY: NS—non-significant; Sig—Significant.

### 3.1. Sleep Latency

Sleep latency is the amount of time (usually in minutes) it takes a person to fall asleep, starting from the first intention to fall asleep. The studies with sleep latency and cognition in older adults have reported mixed results. The results suggest a greater link between sleep latency and global cognition than sleep latency and the particular domains of cognition. Schmutte, *et al.* [17] reported that in a cross-sectional study, while longer sleep latency was associated with worse performance on measures of attention, working memory, verbal fluency and processing speed, after multivariate adjustment, longer sleep latency was associated with verbal fluency only. Findings from three other studies did not indicate a relationship between sleep latency and executive function [18,19], attention [18,19], episodic memory [15,17,18], working memory [18,19,20], verbal fluency [18] and processing speed [18,19]. Three cross sectional studies reported that longer sleep latency was associated with worse performance on global cognition measures [19,21,22], while four other studies [18,23,24,25] reported no relationship between the two variables. When examining objective reports, Blackwell, *et al.* [12] reported that longer sleep latency was associated with worse executive function, attention and global cognition while Miyata, *et al.* [20] did not find any association between sleep latency and attention or working memory.

### 3.2. Wake after Sleep Onset

Wake after sleep onset is total amount of time awake during the night, from the time the person falls asleep until final awakening. There is potential evidence to support the relationship between longer wake after sleep onset and worse domain-specific and global cognition. In two gender-specific studies using actigraphy, wake after sleep onset was associated with worse global cognitive function in both men and women after adjustment for depression and multiple demographic, physical, and health factors [12,13]. Longer wake after sleep onset, derived from the PSQI, was also associated with worse global cognition [21]. However, when using an investigator-developed questionnaire to assess sleep, Keage, *et al.* [23] reported that longer wake after sleep onset was not associated cross-sectionally or longitudinally with global cognitive function. No study examined the association between subjective wake after sleep onset and the domains of cognition; however, longer wake after sleep onset, examined objectively, was associated with worse performance in tests of executive function [12,13,26], attention [12,13], episodic memory [26], and verbal fluency [26] and global cognition [12,13]. In other studies, wake after sleep onset was not associated with attention [20], working memory [20,26] and processing speed [26]. Discrepant finding could be due to using different measures to assess the wake after sleep onset or the cognitive domains.

### 3.3. Sleep Efficiency

Sleep efficiency is the ratio of the time a person is sleeping to the time that is actually spent in bed trying to sleep. The studies with sleep efficiency and cognition suggest that there is a relationship between subjective and objective measured sleep efficiency and global cognition. When looking at the domains of cognition, there appears to be a relationship between the domains and objectively assessed sleep but not subjectively assessed sleep. Using the PSQI, Chang-Quan, *et al.* [21] and Nebes, *et al.* [19] reported that, based on cross-sectional analyses, as sleep efficiency decreased, global cognitive function worsened. Similarly, Potvin, *et al.* [24] reported that longitudinally as sleep efficiency decreased, measured with the PSQI, global cognitive function worsened; however, the association was significant for male but not female participants. Using an investigator-developed questionnaire, Tworoger, *et al.* [15] reported no longitudinal relationship between sleep efficiency and global cognitive function. In objective assessments, there were contrasting results reported by the two Blackwell and colleagues’ studies: in the study with only female participants [12] they found that a relationship was present between the two variables, while in the study with only male participants [13] there was no association between the variables.

As objective sleep efficiency decreased, executive function [12,13,16], attention [12,13,16], working memory [20] and processing speed [16] worsened. There was no relationship between sleep efficiency and attention [20] and verbal fluency [16]. Subjectively measured sleep efficiency was not associated with executive function [16,19], attention [16,19], episodic memory [19], working memory [19], verbal fluency [16] and processing speed [16,19]. Since all the studies were cross-sectional, the difference in the results could be due to different studies using only one measure or multiple measures for the same domain. For example, Nebes, *et al.* [19] used multiple measures to evaluate executive function while the two studies by Blackwell, *et al.* [12,13] each only used one measure.

### 3.4. Sleep Duration

In some of the studies, sleep duration was examined as a continuous variable while in other studies sleep duration was dichotomized into short and long sleep duration. When sleep was examined as a continuous variable, there was no relationship between sleep duration and the domains of and global cognition. However, a U-shaped relationship appeared to emerge when sleep duration was dichotomized and results suggest a relationship between short and long sleep and domains of cognition and global cognition. In a study with older women, Lambiase, *et al.* [16] reported that subjectively measured total sleep duration was associated with executive function and attention but not processing speed; while, objectively measured sleep duration was not associated with any of the cognitive domains. Wilckens, *et al.* [26] had similar results with no association between objectively measured total sleep duration and executive function, episodic memory, working memory and processing speed in a cross-sectional assessment of older adults. Blackwell, *et al.* [13] also found no association between objectively measured total sleep duration and executive function and attention. In subjective assessment, total sleep duration was not associated with executive function [18,19,27], attention [18,19], episodic memory [18,19], working memory [18,19], processing speed [18,19,27] and global cognition [18,19].

In terms of subjective short sleep duration and global cognition, Potvin, *et al.* [24] had mixed results based on gender. Men with short sleep duration had worse global cognition after one year while there was no longitudinal relationship between short sleep duration and global cognition in women. Benito-León, *et al.* [28] reported that there was a cross-sectional association between short sleep and global cognitive function; however, this association was no longer present at the 3-year follow-up. Tworoger, *et al.* [15] had similar results in their cross-sectional and longitudinal (2 years) analyses. In longitudinal analyses, after 22.5 years, Virta, *et al.* [29] found that short sleep duration was associated with poorer cognition. Keage, *et al.* [23] had contrasting results with no association between short sleep and global cognitive function at baseline but an association between short sleep and global cognitive impairment at two and 10 years. Loerbroks, *et al.* [30] found no cross-sectional nor longitudinal association between short sleep duration and global cognitive function. While Blackwell, *et al.* [13], Devore, *et al.* [14], and Xu, *et al.* [31] reported cross-sectional association between subjective short sleep and global cognition, other researchers, Auyeung, *et al.* [22], Ohayon and Vecchierini [32], Ramos, *et al.* [11], Faubel, *et al.* [33] and McCrae, *et al.* [27] had contrasting results.

When looking at the domains of cognition, subjective short sleep duration was associated with attention [30], episodic memory [14,31], working memory [14] and verbal fluency [14]. In contrast, short sleep duration was not related to executive function [13], attention [13,17], episodic memory [15,17,30], working memory [17], verbal fluency [17], and processing speed [17]. Tworoger, *et al.* [15] reported an association between short sleep duration and verbal fluency at baseline, which was no longer significant at follow-up. In objective findings, Miyata, *et al.* [20] reported a relationship between short sleep duration and working memory using the 0-back test. However, using the 1-back test, there was no relationship between the two vaiables. None of the other studies that looked short sleep duration and domains of cogniton found an association [13,20,26]. These studies also did not find associations with objective long sleep duration and the domains of cogniton.

Potvin, *et al.* [24] found that in women but not men, long sleep duration was associated with incident cognitive impairment over one year. Virta, *et al.* [29] found that long sleep was associated with poorer cognition in a 22.5 year follow up of older adults. Benito-León, *et al.* [28] reported that while there was no cross-sectional relationship between long sleep duration and global cognitive function, a relationship emerged between the two variables at the three year follow-up. Ramos, *et al.* [11], Blackwell, *et al.* [13], Auyeung, *et al.* [22], Faubel, *et al.* [33], and Xu, *et al.* [31] reported relationships between long sleep duration and worse global cognitive function; while, Tworoger, *et al.* [15], Keage, *et al.* [23], Ohayon and Vecchierini [32] and Loerbroks, *et al.* [30] reported that long sleep duration was not related to global cognitive function.

### 3.5. General Sleep Problems

There are mixed findings in the relationship between general sleep problems and domains of cognition and global cognition but imply a possible link between increased sleep problems and reduced cognition. In the study by Potvin, *et al.* [24], the sleep quality score in men and the sleep disturbance score in women were associated with global cognition while there was no association between the sleep quality score in women and the sleep disturbance score in men with global cognition. Tworoger, *et al.* [15] reported that there was a cross-sectional but not longitudinal relationship between general sleep problems and global cognition. Saint Martin, *et al.* [18] reported that the global PSQI score was associated with worse global cognition; while, the PSQI sleep quality score was not associated with global cognition. Auyeung, *et al.* [22] revealed that in univariate analyses sleep problems were associated with global cognition but were no longer associated after multivariate analyses. Nebes, *et al.* [19], Sampaio, *et al.* [34], Virta, *et al.* [29] and Chang-Quan, *et al.* [21] reported that there was a relationship between subjective general sleep problems and global cognition and Lim, *et al.* [35] reported that there was a relationship between objective general sleep problems and global cognition. In contrast, Blackwell, *et al.* [13], Keage, *et al.* [23], Jaussent, *et al.* [25], Foley, *et al.* [36], Gamaldo, *et al.* [37] and Zimmerman, *et al.* [38] reported no associations between subjectively measured general sleep problems and global cognition.

There are conflucting results for general sleep problems and each of domains of cognition. Saint Martin, *et al.* [18], Nebes, *et al.* [19], Gamaldo, *et al.* [37], Sutter, *et al.* [39] and McCrae, *et al.* [27] all reported an association with general sleep complaints and at least one of the domain of cognition while Saint Martin, *et al.* [18], Nebes, *et al.* [19], Sutter, *et al.* [39] and McCrae, *et al.* [27] in addition to Zimmerman, *et al.* [38], Blackwell, *et al.* [13], Miyata, *et al.* [20] and Tworoger, *et al.* [15] reported that there was no relationship between the variables.

## 4. Discussion

The aim of the review was to understand the relationship between sleep parameters and the domains of cognition and global cognition in adults 60 years and older. In this population, the research on the association of subjective sleep parameters and cognition is mixed; as a result, more studies, particularly longitudinal studies, are needed that further explores the relationship among these variables. Interestingly, sleep duration, a sleep variable most consistently related to disease states such as cardiovascular disease [44,45], was not consistently associated with changes in cognitive function. A recent review suggests that older adults may actually be more resistant to the cognitive effects of sleep problems, such as deprivation and restriction [46] possibly due to physiologic adaptation throughout the aging process.

A majority of the studies used available data as partial measures of sleep, as many of these studies were secondary analyses. These measures often were not validated or demonstrated to be reliable and thus cannot be readily compared against other valid and reliable measures of sleep. Even in studies with good measures of sleep, the measure primarily reflected an overarching score of sleep. For instance, the PSQI assesses sleep with a single score reflecting overall sleep in the last month. In addition, sleep parameters were assessed differently by different questionnaires. For example, sleep latency was assessed by asking the participants to indicate the number of minutes taken to fall asleep or by asking if they usually took long to fall asleep. There was also a lack of standardization of the cut-off times for some of the sleep variables such as sleep latency and sleep duration. For example, Ohayon and Vecchierini [32] used short sleep duration as <7 h and long sleep duration as >8.5 h while Loerbroks, *et al.* [30] defined short sleep duration as <6 h and long sleep duration as >9 h. Future studies should attempt to standardize the cut-points used for long and short sleep duration. Another limitation is the use of subjective sleep measures in many studies of cognitive function. Subjective measures can possibly lead to differential misclassification and selective drop-out, because persons with poor cognitive function are likely to have more difficulty to accurately complete sleep questionnaires and sleep diaries.

Objective sleep measures are necessary, but have their own strengths and weakness. For example, actigraphy provides a plethora of activity data over longer periods of time but is often not sensitive to time spent awake lying still and does not measure sleep stage [47]; whereas polysomnography (PSG) uses many physiologic measures to capture sleep and sleep parameters but is often only completed on one or two nights [48]. Sleep in older adults is quite variable and the influence of that factor was generally not accounted for in the reviewed studies. Examining the relationship between night to night sleep variability using multi-night objective measures of sleep is necessary to further assess the relationship between objective sleep and cognition.

Sleep architecture parameters specific to rapid eye movement (REM) and non-rapid eye movement (nREM) sleep were not examined in this review. Since older adults report increase in sleep fragmentation and more time in lighter sleep stages [49], it is possible that these parameters are the ones that are more associated with changes in cognitive function. In order to determine if there is an association, PSG needs to be used on a more consistent basis. Home polysomnography is now an option and may be better and more convenient for the participants.

The inconsistency of measurement of cognition and sleep also limits the ability to identify relationships across the reviewed studies. First, there was variation across the studies in the assessment measures for sleep and cognitive function. Although the measures used for cognitive function in the majority of studies were valid and reliable, the same measure was not consistently used by the researchers to examine global or domains of cognition. For example, Trail Making B, Stroop Color and Word test, Oral Word Fluency test, Porteus Maze, and Optimal Telegram were all used to assess executive function. As pointed out by Snowden, *et al.* [50], it would be beneficial if there was a consensus of measures, such as the National Institutes of Health Toolbox or the Uniform Data Set of the Alzheimer’s Disease Center, to allow for better comparison across studies. Another limitation within the cognitive domain involves the measures used to assess global cognitive functioning. Many of the measures, like the MMSE, may not be sensitive enough to identify small but significant changes in cognition.

Additionally, there are important covariates that must be accounted for in studying the relationship between sleep parameters and cognition. Depression is a common cause of sleep problems in older adults and associated with neurocognitive impairments such as slower processing speed and executive dysfunction [51]. Aloia, *et al.* [52], Steffens, *et al.* [53] and Zimmerman, *et al.* [38] have also posited that depression and depressive symptoms are associated with a decline in cognitive function. In their review, Foley, *et al.* [36] reported that sleep problems did not predict cognitive decline after controlling for depression; however, depression at baseline significantly increased the likelihood of a decline in cognitive function at follow-up. Schmutte, *et al.* [17] reported that sleep latency and total sleep time were moderately related to depression and Saint Martin, *et al.* [18] reported that subjective evaluation of cognitive function was related to the depression score. Nebes, *et al.* [19] pointed out that the participants who reported poor sleep had more depressive symptomatology than those reporting good sleep; therefore, it is possible that poorer sleep was related to depression which then contributed to poorer cognitive functioning for that specific group of older adults.

Another alternative explanation is that study participants with undiagnosed sleep apnea may have contributed to the inconsistency in the association between sleep parameters and cognitive function. Most studies in the review did not screen for or ask about a sleep apnea diagnosis or had sleep apnea as a confounder, so did not account for the possible confounding effect of the presence of sleep apnea. Sleep apnea is associated with worse verbal fluency and constructional tasks [52] and without a screen or diagnosis, it is challenging to adjust for the presence of the disorder or symptoms.

Both age and poorer health appear to play a role in the relationship between sleep and cognition. Blackwell, *et al.* [12], Chang-Quan, *et al.* [21] and Lim, *et al.* [35] all reported that participants with a mean age over 80 years old reported that the worse the sleep parameters, the worse their cognitive function measures. Denton and Spencer [54] reported that in the oldest old population, the prevalence rate and the relative prevalence of chronic conditions such as dementia, stroke, and heart disease were much higher for persons over 80 years than for persons under age 80. Wolff, *et al.* [55] also reported than adults over 80 years were more likely to have more than four chronic illnesses compared to their younger counterparts. Kronholm, *et al.* [56] reported that the relationship between sleep and cognitive function disappeared when they accounted for the participants’ health status.

## 5. Conclusions

The current research on the association of subjective sleep parameters and cognition is inconclusive and there is insufficient evidence to confirm or deny the existence of a relationship between objective sleep parameters and cognition. Primary studies using valid and reliable measures for all sleep and cognitive variables are clearly needed; it would be very useful if similar measures were used across studies. Future research should also account for important covariates such as depression, obstructive sleep apnea, age and chronic medical illness. Healthcare providers should be aware that sleep disturbances can be associated with cognition in older adults and that medical and psychiatric conditions can influence the association. With this knowledge, providers should perform baseline and periodic ongoing assessments to identify changes in sleep patterns, cognition, and the risk factors that can influence the association between sleep and cognition.
